# Assessing Total Fluoride Intake in Children: Reliability of Commonly Used Methods

**DOI:** 10.1111/cdoe.70015

**Published:** 2025-08-21

**Authors:** Fatemeh Vida Zohoori, Marilia Afonso Rabelo Buzalaf, Anne Maguire, Roy Sanderson, Rodrigo A. Giacaman, Stefania Martignon, Edgar O. Beltran, Fatemeh Eskandari, Jelena Kronic, Karla Gambetta‐Tessini, Flavia Mauad Levy

**Affiliations:** ^1^ School of Health and Life Sciences Teesside University Middlesbrough UK; ^2^ Bauru School of Dentistry University of Sao Paulo Sao Paulo Brazil; ^3^ School of Dental Sciences, Faculty of Medical Sciences Newcastle University Newcastle UK; ^4^ School of Natural & Environmental Sciences Newcastle University Newcastle UK; ^5^ Cariology Unit, Department of Oral Rehabilitation, Faculty of Dentistry University of Talca Talca Chile; ^6^ UNICA – Caries Research Unit, Research Department Universidad El Bosque Bogotá Colombia; ^7^ National Dental Research Institute National Dental Centre Singapore Singapore

## Abstract

**Objectives:**

Fluoride exposure in children is commonly estimated using questionnaires or urinary biomarkers. However, no study has yet compared these methods for classifying participants into five intake categories ranging from low to high. This study aimed to estimate the extent of agreement and classification consistency between questionnaire‐ and urinary‐based methods for assessing total daily fluoride intake (TDFI) in children aged 4–7 years.

**Methods:**

A total of 104 healthy children across three countries (UK, Brazil, Chile) receiving one of three fluoridation modalities (non‐fluoridated‐water, fluoridated‐water, or fluoridated‐milk) provided a 24‐h urine sample and completed validated dietary and oral hygiene questionnaires. TDFI was estimated from dietary sources and toothpaste ingestion, adjusted for body weight. Urinary fluoride concentration was measured and 24 h‐UFE determined by multiplying urine volume by fluoride concentration. TDFI was predicted from 24 h‐UFE using the WHO's recommended method. Method agreement was assessed using paired t‐tests and Bland–Altman analysis to evaluate continuous fluoride intake estimates. Cohen's kappa was used to assess agreement between categorical intake classifications, while descriptive statistics reported the percentage of children in each intake group.

**Results:**

The questionnaire method estimated a higher mean TDFI (0.072 mg/kgbw/day) than the urine‐based method (0.058 mg/kgbw/day, *p* = 0.01). Bland–Altman analysis showed good agreement for lower mean TDFI values (< 0.10 mg/kgbw/day) but increasing variability at higher fluoride intake levels. The questionnaire classified a larger proportion of children as high exposure (≥ 0.1 mg/kgbw/day) than the urine method (19.2% vs. 11.5%), with the greatest discrepancy observed in the fluoridated milk group (46.2% vs. 7.7%). Despite these classifications, Cohen's kappa revealed minimal agreement between methods (κ = 0.034, *p* = 0.508), suggesting that classification concordance was likely due to chance.

**Conclusion:**

This first study comparing questionnaire and urinary methods for assessing TDFI in children found significant discrepancies and minimal agreement, especially in higher exposure groups, highlighting the risk of misclassification and the need for research into combined assessment approaches.

## 1 Introduction

Fluoride is widely recognised for its role in preventing dental caries, making it a key component of public health strategies aimed at promoting oral health in children. While low concentrations of fluoride help prevent dental caries by strengthening enamel and promoting tooth remineralisation, excessive fluoride intake, even over short periods during tooth formation, can increase the risk of developing dental fluorosis, which may result in unsightly tooth discolouration. Therefore, it is essential that total fluoride intake from all sources does not exceed the Tolerable Upper Intake Level (0.1 mg/kg body weight (bw)/day) during the first 5–8 years of life to minimise the risk of dental fluorosis [1].

Consequently, accurate measurement of fluoride intake from all potential sources, including diet and accidental ingestion of toothpaste during brushing, is essential, although it remains both challenging and resource‐intensive [2]. Total daily fluoride intake (TDFI) is commonly assessed through diet and oral hygiene questionnaires or by measuring 24‐h urinary fluoride excretion (24 h‐UFE), which serves as a recent biomarker of total fluoride intake, particularly in children [3]. However, both methods have inherent limitations that may affect their applicability depending on the population or exposure route.

Urinary fluoride excretion, particularly through 24‐h urine samples, provides an estimate of systemically absorbed bioavailable fluoride. Since the relationship between 24 h‐UFE and TDFI in children and adults has been numerically modelled, TDFI can be quantitatively estimated from 24 h‐UFE measurements using the published numerical relationship [3]. However, fluoride excretion via urine can be influenced by several factors, including diet. For instance, a vegetarian diet, which tends to make urine more alkaline, results in higher fluoride excretion compared to a diet high in meat [4]. Additionally, this method does not identify the specific sources of fluoride intake. Collecting 24‐h urine samples also presents significant practical challenges, particularly in young children, especially those who are not toilet trained, making this approach less feasible in many settings.

Conversely, using questionnaires to estimate TDFI is less burdensome for study participants and can capture detailed information on the various sources of fluoride exposure, including dietary intake and toothpaste use. However, this method is susceptible to reporting bias, as participants may underreport or overreport their intake. Furthermore, it is labour‐intensive for researchers, as it requires coding and analysis of the fluoride content of individual food and beverage items consumed, often involving complex and time‐consuming procedures.

There is no robust evidence to suggest any advantage in one method over another for the assessment of fluoride intake, and the choice often depends on the objectives of investigation and the population being studied. Although several studies [2, 3] have investigated the correlation between fluoride intake and excretion, none have directly compared the number or proportion of participants classified into different fluoride intake categories using these two methods. This comparison is crucial, as misclassifying fluoride intake categories of participants could compromise the reliability of the findings and undermine the validity of conclusions regarding fluoride exposure and its associated health effects.

Therefore, the aim of this study was to estimate the extent of agreement and classification consistency between questionnaire‐ and urinary‐based methods for assessing total daily fluoride intake (TDFI) in children aged 4–7 years.

## 2 Material and Methods

Ethical clearance was obtained from the relevant institutional committees in the UK (#007/19, Oct/2019), Brazil (# CAAE 12565319.9.0000.5417) and Chile (#05‐2020E, Sept/2023). Written informed consent was obtained from all the parents of the children recruited before the study was initiated.

Healthy children aged 4–7 years, who were lifelong residents of the study area, free from metabolic or renal diseases, not using systemic fluoride and without receiving professionally applied topical fluoride treatments (e.g., varnishes or gels) in the past month, were invited to participate in the study. Participants were recruited through convenience sampling due to the practical challenges of randomised sampling in studies involving young children.

A total of 150 children were recruited, with 30 children from each of five locations representing different fluoridation modalities across three countries (Table 1). The selection of these locations was purposive, aiming to capture diverse fluoride intake, including variations in dietary intake and oral hygiene habits, relevant to the study objectives. The sample size of 30 per site was based on World Health Organisation (WHO) recommendations for 24‐h urine collection studies in community prevention programmes, which suggest recruiting approximately 30 participants per site [5]. While a previous study [6] indicated that a total sample size of 22 would provide 80% power to detect correlations between TDFI and 24 h‐UFE at *p* = 0.05, the larger per‐site sample size was chosen to improve representativeness and statistical robustness across varied fluoridation settings.

**TABLE 1:** Study locations and fluoridation modalities.

Location	Fluoridation modality	Code
UK, Middlesbrough	Non‐fluoridated Water (< 0.3 mgF/L of drinking water)	NFW‐UK
Chile, San Clemente, Maule Region	Non‐fluoridated Water (< 0.3 mgF/L of drinking water)	NFW‐Chile
UK, Hartlepool	Fluoridated Water (0.8–1.3 mgF/L of drinking water)	FW‐UK
Brazil, Bauru	Fluoridated Water (0.8 mgF/L of drinking water)	FW‐Brazil
Chile, San Clemente and San Javier	Fluoridated Milk (4.25 mgF/L of powdered milk)	FM‐Chile

A standardised protocol for data and sample collection, including urine sampling and questionnaire administration, was developed collaboratively by the research teams and subsequently implemented across all sites. All teams received training prior to data collection, and regular communication was maintained throughout the study to ensure consistency between sites and resolve any issues.

### 2.1 Collection of Samples and Data

Samples and data were collected over two separate visits. At Visit 1, children's weight was measured to the nearest 0.1 kg using a portable digital scale, with shoes and jackets removed. Parents were given collection containers and bottles, along with both written and verbal instructions for data and sample collection. Additionally, a tap water sample was collected from each child's home.

#### 2.1.1 Intake Data

During the first visit, parents of children were interviewed about their child's toothbrushing habits/routine using a validated, standardised, interviewer‐administered questionnaire [7], which included a pictorial scale of the amount of toothpaste routinely used.

Dietary data were collected using 24 h dietary recall (24H‐R) [8]: parents were provided with a self‐administrated 24H‐R form and accompanying instructions to record all food and drink consumed by their child over a 24 h period. The 24H‐R was structured to capture detailed information about all foods and beverages consumed by the respondent in the past 24 h. A postcompletion interview with parents was conducted on the second visit to ensure that all dietary data had been recorded as precisely as possible.

#### 2.1.2 Urine Samples

At Visit 1, parents were instructed to collect a single complete 24‐h urine sample from their child, which involved collecting all urine voided over a full 24‐h period into provided containers. Parents received both written and verbal instructions, and collection containers were labelled and coded. The collected samples were picked up at Visit 2 and transported to the Fluoride Laboratory at each respective institute in the participating countries, where their volumes were measured. Aliquots of the urine were stored in a freezer at –18°C until fluoride analysis.

The completeness of the 24‐h urine collections was assessed by evaluating urinary flow rate against WHO criteria for validating urine data in children aged 4–6 years. A flow rate of less than 7 mL/h is considered indicative of incomplete urine collection [5].

### 2.2 Assessment of Fluoride Concentration

Fluoride concentrations of urine and water samples were measured in triplicate at room temperature using F‐ion‐selective electrode (F‐ISE Model 79 609; Orion Research) coupled to a potentiometer (Model 720A) and a direct F analysis method after adding TISAB III [9].

Additionally, Fluoride Urine Standard Reference Materials (FU‐SRM1805 and FU‐SRM1815) from the Institut National de Santé Publique du Québec, Canada, were analysed to ensure the validity and consistency of the analytical methods across participating laboratories. Additionally, the reliability of the analytical methods was confirmed by reanalysing 10% of the samples. All analyses, including initial testing and reanalysis, were performed in triplicate.

### 2.3 Data Handling

#### 2.3.1 Questionnaire‐Derived TDFI (TDFI‐Q)

Fluoride intake from diet (mg/day) was calculated by combining fluoride intake from all consumed food and drinks by each child. All recorded food and drink items, along with their portion sizes (g), were entered into an Excel file. Fluoride databases [10, 11] were then used to calculate the fluoride content of each item individually by multiplying the weight of the item (g) by its fluoride concentration (mg/g). These individual values were combined to determine the total daily dietary fluoride intake (mg/day).

Fluoride ingestion from dentifrice (mg/day) was estimated by combining the frequency of tooth brushing, the fluoride concentration of the reported toothpaste brand, the amount of dentifrice used and the estimated percentage of toothpaste that was swallowed. The quantity of toothpaste used and swallowed was estimated by having parents select pictures of toothbrushes with varying amounts of toothpaste in the interviewer‐administered questionnaire.

TDFI (mg/day) was calculated by combining fluoride intake from both the diet and toothpaste ingestion.

#### 2.3.2 Urinary Excretion‐Based Predicted TDFI (TDFI‐U)

The 24‐h urinary fluoride excretion (24 h‐UFE, μg/24 h) was calculated by multiplying the volume of each urine sample (ml/24 h) by its fluoride concentration (μg/ml).

TDFI was estimated from 24 h‐UFE using a predictive equation recommended by the WHO for assessing fluoride intake in community oral health programmes [5]:

DUFE = (TDFI × 0.35) + 0.03.

This equation was developed in a comprehensive study using paired intake and urinary excretion data from 212 children across multiple countries and locations, modelling the relationship between fluoride intake and urinary excretion in children [3].

To calculate TDFI from measured DUFE values, the equation was rearranged as follows:

TDFI = (DUFE–0.03) / 0.35, where TDFI and DUFE are expressed in mg/24 h.

#### 2.3.3 Adjusting TDFI by Body Weight

Both TDFI‐Q and TDFI‐U were adjusted for body weight (mg/kg bw/day) by dividing the total daily fluoride intake (TDFI, mg/day) by the child's weight (kg).

#### 2.3.4 Classification of Individuals Into Low, Intermediate and High Fluoride Intake Groups

Fluoride intake was classified into low, intermediate (below‐moderate, moderate and above‐moderate) or high exposure categories based on WHO recommendations [5]. Low exposure was defined as ≤ 0.02 mg/kg bw/day, and high exposure as ≥ 0.1 mg/kg bw/day, reflecting WHO [5] and European Food Safety Authority [12] thresholds for minimal and elevated risk respectively. Intake levels between these thresholds were further stratified into three intermediate bands: below‐moderate (> 0.02–< 0.05 mg/kg bw/day), moderate (0.05–0.07 mg/kg bw/day) and above‐moderate (> 0.07–< 0.10 mg/kg bw/day), based on WHO‐defined intake ranges [5] to allow for more detailed analysis of potential dose–response relationships.

## 3 Statistical Analysis

Data were analysed descriptively using SPSS. A paired t‐test was conducted to compare the two assessment methods at the group level. Agreement between continuous fluoride intake estimates derived from the questionnaire and urinary measurements was assessed using Bland–Altman analysis, which involved calculating the mean difference and the limits of agreement (mean difference±1.96 standard deviations). Agreement between fluoride exposure classifications based on WHO intake thresholds [5] was further evaluated using Cohen's kappa statistic to assess categorical concordance. Cohen's kappa was calculated using unweighted agreement.

## 4 Results

### 4.1 Quality Control of Fluoride Analysis

The overall mean (SD) fluoride concentration of FU‐SRM1805 was 0.384 ± 0.003 mg/L, and for SRM‐1815, it was 0.613 ± 0.008 mg/L. Both values fell within their respective certified ranges (0.366–0.390 mg/L for FU‐SRM1805 and 0.601–0.628 mg/L for SRM‐1815), confirming the validity and consistency of the analytical method across laboratories.

Analytical accuracy was further supported by reanalysis of 10% of the samples. No statistically significant difference was observed between the initial and repeated measurements (*p* = 0.426), with a mean difference of 0.003 ± 0.02 mgF/L, indicating high reproducibility of the method.

### 4.2 Study Participants

Out of the 150 children recruited, 104 provided both a 24‐h urine sample and completed a 24‐h dietary recall and oral hygiene questionnaire. The remaining 46 children were excluded due to incomplete dietary records, incomplete urine collections (including issues such as bedwetting at night and refusal by the child to provide urine samples at certain times), or missing child weight data. The mean age of the excluded children was 5.2 years, and their mean weight was 20.8 kg.

The mean urinary flow rate among the 104 children who provided urine samples was 24.4 mL/h, with a range of 8.6–45.1 mL/h. As all values exceeded the minimum threshold for completeness, no participants were excluded from the analysis. The overall mean (SD) age of the children was 5.6 (0.8) years, and their mean weight was 23.4 (6.3) kg (Table 2).

**TABLE 2:** Mean (SD) age and weight of children who provided both a 24‐h urine sample and exposure questionnaires.

Fluoridation modality	Number of participants	Mean (SD)	
		Age (years)	Weight (kg)
NFW‐UK	12	5.1 (0.8)	19.0 (3.3)
NFW‐Chile	27	5.6 (0.8)	24.2 (6.0)
FW‐UK	18	5.5 (0.9)	21.9 (3.9)
FW‐Brazil	21	5.0 (0.9)	20.0 (4.6)
FM‐Chile	26	6.9 (0.6)	28.4 (6.8)
All	104	5.6 (0.8)	23.4 (6.3)

Abbreviations: FM, fluoridated milk; FW, fluoridated water; NFW, Non‐fluoridated water.

### 4.3 Comparison of Total Daily Fluoride Intake Using the Two Methods

**FIGURE 1:**
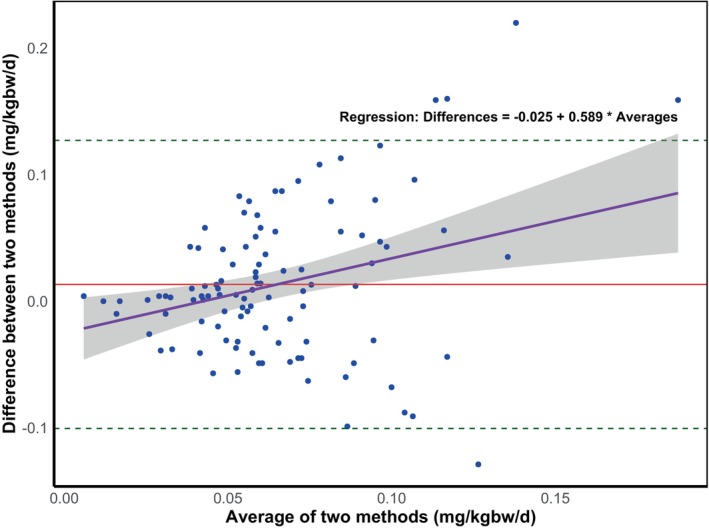
Bland–Altman plot where the dashed lines represent the 95% upper and lower limits of agreement, the middle horizontal line represents the mean difference between the two methods, the regression line indicates the trend in differences and the shaded area around the regression line indicates the 95% confidence interval for the regression line.

The Bland–Altman plot for overall mean differences in TDFI estimated by the two methods (TDFI‐Q and TDFI‐U) is presented in Figure 1. The plot shows good overall agreement between the two methods in estimating TDFI but also indicates proportional bias. Agreement was very good for lower mean values (up to approximately 0.10 mg/kg bw/day), with variability increasing as the mean values rose. The mean difference between the two methods was 0.013 mg/kg bw/day, indicating a slight overestimation by the questionnaire method. However, the limits of agreement were wide, ranging from −0.101 to +0.127 mg/kg bw/day, reflecting substantial variability in individual differences between the methods.

Table 3 presents the results of the paired t‐test for TDFI estimated using TDFI‐Q and TDFI‐U for combined data across all modalities, as well as for each individual fluoridation modality. The overall mean TDFI estimated by questionnaire (0.072 mg/kgbw/day) was significantly higher (*p* = 0.01) than that estimated by urine collection (0.058 mg/kgbw/day). However, when examining each fluoridation modality individually, the difference observed between the two methods was highly significant for fluoridated milk (*p* < 0.001) and moderately significant for Brazil fluoridated water (*p* = 0.01), with no significant difference for the other modalities.

**TABLE 3:** Comparison of mean TDFI (mg/kgbw/day) estimated by Questionnaire‐derived TDFI (TDFI‐Q) and Urinary excretion‐based predicted TDFI (TDFI‐U).

Fluoridation modality	No.	Mean (SD)		Difference		
		TDFI‐Q	TDFI‐U	Mean (SD)	95% CI	*P* a
NFW‐UK	12	0.055 (0.047)	0.042 (0.030)	+0.011 (0.044)	−0.016, +0.039	0.192
NFW‐Chile	27	0.061 (0.031)	0.049 (0.029)	+0.012 (0.040)	−0.004, +0.028	0.069
FW‐UK	18	0.073 (0.044)	0.089 (0.041)	−0.017 (0.068)	−0.051, +0.017	0.157
FW‐Brazil	21	0.046 (0.011)	0.060 (0.027)	−0.014 (0.026)	−0.026, −0.002	0.01
FM‐Chile	26	0.110 (0.057)	0.051 (0.031)	+0.059 (0.065)	+0.032, +0.085	< 0.001
All children	104	0.072 (0.047)	0.058 (0.034)	+0.013 (0.058)	+0.002, +0.025	0.01

Abbreviations: FM, fluoridated milk; FW, fluoridated water; NFW, Non‐fluoridated water.

^a^ Based on paired *t*‐test.

#### 4.3.1 Comparison of the Percentage of Children With Low and High Fluoride Intake at Individual Level Using the Two Methods

**FIGURE 2:**
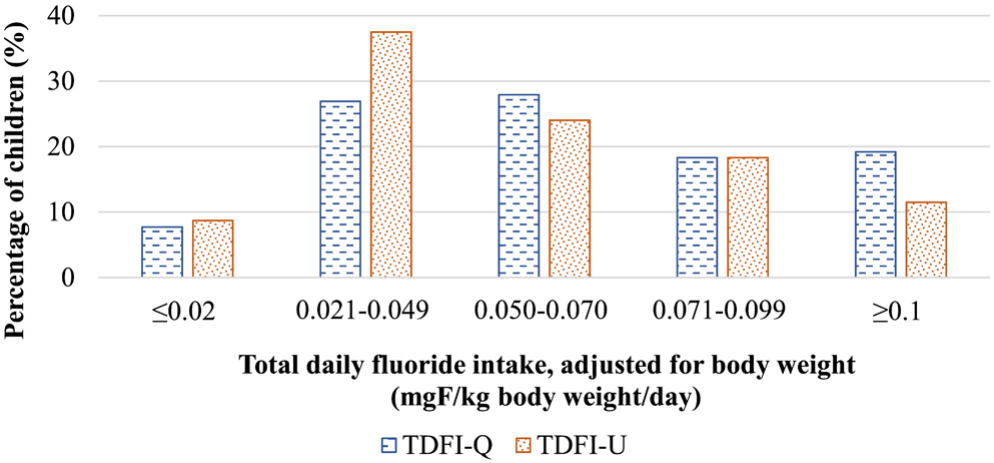
Distribution of total daily fluoride intake (TDFI) levels in all children (*n* = 104) according to method of data collection: questionnaires (TDFI‐Q) and urine collection (TDFI‐U).

Figure 2 presents the distribution of children according to fluoride intake levels, as estimated by the TDFI‐U and TDFI‐Q methods. The majority of children (73% based on TDFI‐Q and 80% based on TDFI‐U) had fluoride intake levels between the low exposure (≤ 0.02 mg/kgbw/day) and high exposure thresholds (≥ 0.1 mg/kgbw/day).

Table 4 presents the percentage of individuals classified into different exposure categories for different fluoridation modalities, based on TDFI estimates from both questionnaire and urine‐based methods.

**TABLE 4:** Percentage of children with estimated low, intermediate (below‐moderate, moderate, above‐moderate) and high fluoride exposure according to method; questionnaire (TDFI‐Q) and urine collection (TDFI‐U).

Fluoridation modality	Total daily fluoride intake categories (mg/kgbw/day) by method									
	Low (≤ 0.02)		Below‐moderate (> 0.02 ‐ < 0.05)		Moderate (0.05–0.07)		Above‐moderate (> 0.07 ‐ < 0.1)		High (≥ 0.1)	
	TDFI‐Q	TDFI‐U	TDFI‐Q	TDFI‐U	TDFI‐Q	TDFI‐U	TDFI‐Q	TDFI‐U	TDFI‐Q	TDFI‐U
NFW‐UK (*n* = 12)	25.0	16.7	33.3	41.7	8.3	33.3	16.7	0.0	16.7	8.3
NFW‐Chile (*n* = 27)	11.1	14.8	25.9	44.4	22.2	25.9	33.3	7.4	7.4	7.4
FW‐UK (*n* = 18)	5.6	0.0	27.8	5.6	33.3	27.8	11.1	38.9	22.2	27.8
FW‐Brazil (*n* = 21)	4.8	4.8	52.4	38.1	42.9	23.8	0.0	23.8	0.0	9.5
FM‐Chile (*n* = 26)	0.0	7.7	3.8	50.0	26.9	15.4	23.1	19.2	46.2	7.7
All (*n* = 104)	7.7	8.7	26.9	37.5	27.9	24.0	18.3	18.3	19.2	11.5

Abbreviations: FM, fluoridated milk; FW, fluoridated water; NFW, Non‐fluoridated water.

For the combined data across all fluoridation modalities, the questionnaire‐based method indicated a higher overall percentage of individuals in the ‘high exposure’ category (19.2%) compared to the urine‐based method (11.5%). However, the most notable difference was observed in the fluoridated‐milk modality, where 46.2% of individuals were classified in the high exposure category by the questionnaire method, compared to just 7.7% by the urine‐based method.

Agreement between fluoride exposure classifications derived from the questionnaire and urinary measurements was assessed using Cohen's kappa. The analysis showed minimal agreement between the two methods (κ = 0.034, *p* = 0.508), indicating that any observed concordance was likely due to chance.

## 5 Discussion

This study compared total daily fluoride intake, estimated using two methods: a validated fluoride exposure questionnaire (TDFI‐Q) and a 24‐h urine sample as a biomarker of fluoride exposure (TDFI‐U). Additionally, for the first time, this study examined the agreement between these two methods in assessing fluoride intake levels in children.

Overall, the paired t‐test showed that the results of the two methods (TDFI‐Q and TDFI‐U) were statistically significantly different, but the difference was minimal (+0.013 mgF/kgbw/day) and the small mean difference is unlikely to have a significant clinical impact for both non‐fluoridated and fluoridated water modalities. This is supported by the tight clustering of data points near the bias line (< 0.10 mgF/kg bw/day) on the Bland–Altman plot (Figure 1). These findings suggest that at the group or community level, the two methods provide broadly comparable estimates of fluoride intake. However, for the milk‐fluoridation modality, the difference in estimated total fluoride intake between the two methods was highly significant (*p* < 0.001), with the questionnaire method estimating nearly twice the intake (0.110 mgF/kg bw/day) compared to the urine‐based method (0.051 mgF/kg bw/day) (Table 3).

When comparing the percentage of children classified into different fluoride exposure levels across all modalities, discrepancies were observed between the two estimation methods, suggesting that the methods assess fluoride intake differently depending on the modality and may lead to inconsistencies in exposure classification. Both Cohen's kappa and Bland–Altman analyses revealed important limitations in the agreement between questionnaire‐based and urinary‐based fluoride intake assessments. Cohen's kappa indicated only slight agreement between the two methods (κ = 0.034, *p* = 0.508), suggesting that discrepancies in classification are largely attributable to chance. Similarly, the Bland–Altman analysis showed a small mean difference (0.013 mg/kg bw/day), but wide limits of agreement (−0.101 to +0.127 mg/kg bw/day), indicating substantial variability at the individual level. Together, these findings raise concerns about the reliability and consistency of fluoride intake classification, as a child's exposure status and the resulting clinical or public health decisions could vary significantly depending on the assessment method used. These results underscore that the two methods are not interchangeable for estimating fluoride intake at the individual level. This emphasises potential limitations in one or both methods and the need for careful consideration when interpreting fluoride exposure estimates.

Overall, the percentage of children classified with low fluoride exposure (≤ 0.02 mg/kgbw/day) was very similar between the two methods (TDFI‐Q: 7.7%, TDFI‐U: 8.7%), confirming general agreement when fluoride intake is low. However, a notable discrepancy was observed in the classification of high fluoride exposure (≥ 0.1 mg/kgbw/day), with the questionnaire method estimating a substantially higher proportion of children with high intake (19.2%) compared to the urine‐based method (11.5%).

Differences in methods for categorising children in the high‐exposure group were particularly evident across different fluoridation modalities. In fluoridated water areas (FW‐UK and FW‐Brazil), urine‐based estimates identified more children with high fluoride exposure than the questionnaire method (Table 4). This suggests that urinary biomarkers may more accurately capture fluoride intake from water. Self‐reported questionnaires rely on recall, which can lead to underreporting due to difficulties in remembering all water consumption or accurately estimating intake volumes. Additionally, the potential over‐ or underestimation of fluoride intake from toothbrushing should be considered, as questionnaires may not fully reflect changing fluoride exposure patterns due to evolving oral hygiene recommendations, such as the widespread adoption of the ‘spit, don't rinse’ guidance on fluoride toothpaste packaging. Further research is needed to better differentiate fluoride intake from drinking water, diet and oral hygiene practices.

In contrast, in the fluoridated milk group, the questionnaire method classified 46.2% of children as having high fluoride exposure, whereas the urine‐based method classified only 7.7%. Moreover, none of the children in this group were classified as having low exposure by the questionnaire method, compared to 7.7% based on the urine‐based method. These discrepancies suggest a systematic overestimation of fluoride intake by the questionnaire method across the range of exposures in this subgroup. This difference could be attributed to children not consistently consuming their entire portion of fluoridated milk, particularly when unsupervised (e.g., in playgrounds) [13]. Despite this, their intake may have been reported as complete, either because they believed they had consumed it or because they shared it with peers, leading to overestimation in the questionnaire‐based assessment.

Children in the fluoridated‐milk group in Chile were slightly older than those in other modalities. Age can influence dietary patterns, as five‐year‐olds typically rely on parental guidance for food choices, whereas seven‐year‐olds have developed greater independence and are more exposed to external dietary influences. However, previous studies [7, 14, 15] on fluoride intake have shown that mean total daily fluoride intake from foods, beverages, dentifrice and supplements generally remains stable from ages 3 to 7 years. Moreover, no known age‐related differences exist in the qualitative aspects of fluoride metabolism, that is, absorption, distribution, utilisation and excretion [16]. While quantitative differences in fluoride metabolism, such as the rate of absorption and excretion, exist between children and adults, they are not observed among children [16]. In this study, total daily fluoride intake was adjusted for body weight to account for any possible age‐related differences, including variations in weight.

Several factors, including diet composition and gastric emptying rate, influence fluoride absorption, thereby affecting the relationship between intake and excretion [17]. Among dietary components, cations such as calcium play a crucial role in reducing fluoride absorption, subsequently lowering its excretion. Milk, with its high calcium content, has been reported to decrease fluoride absorption by up to 13% [18]. Additionally, a recent in vitro study found that the mean fluoride bioavailability for meals consumed with milk dropped to 71.5% [19]. Thus, the large discrepancy observed in this study between the questionnaire and urine‐based methods for the milk–fluoridation modality may also be attributed to differences in total fluoride intake and also the proportion of fluoride in the milk absorbed and excreted. Since the questionnaire method estimates total intake, whereas the urine‐based method reflects only absorbed fluoride, this finding underscores the potential impact of bioavailability on fluoride metabolism and excretion.

For assessing fluoride exposure in children, both questionnaires and 24‐h urine collections can be valuable tools, each with its strengths and limitations. Urinary fluoride provides a direct biological measure of fluoride intake, but it can be influenced by factors unrelated to long‐term intake, such as diet, nutritional status, physical activity, renal function and acid–base imbalances like acidosis [17]. While urinary fluoride is considered a good biomarker for total fluoride exposure, it primarily reflects recent intake, especially in cross‐sectional studies, which capture a snapshot of current exposure rather than chronic exposure. Since urinary fluoride reflects recent exposure, it is less effective for assessing long‐term trends or cumulative exposure. However, it is particularly useful for evaluating recent changes in intake, as it responds quickly to fluctuations in exposure.

On the other hand, questionnaires are useful for capturing long‐term trends in fluoride exposure and are especially valuable for large‐scale studies aimed at assessing population‐level exposure. They are well suited for estimating habitual exposure, particularly in relation to dental fluorosis development, as they capture long‐term intake patterns. They are essential for identifying specific sources of fluoride exposure, such as drinking water, food and other environmental factors. However, it is important to recognise that even a valid estimation method reflects only the period during which it was applied, which may not correspond to the biologically relevant window of exposure. Questionnaires are subject to recall bias and do not directly measure the amount of fluoride absorbed into the body.

Dental caries remains a significant global public health concern, particularly among children and disadvantaged populations. While fluoride is essential for preventing dental decay, excessive fluoride exposure can increase the risk of developing dental fluorosis. This potential side effect underscores the need to carefully balance fluoride exposure to maximise its benefits in preventing caries while minimising the risk of fluorosis, making total fluoride exposure a critical metric to monitor. Additionally, any cause‐and‐effect study should employ precise exposure measurement and rigorous methodologies to assess the potential health impacts of fluoride.

A combined approach using both methods may be ideal for more comprehensive assessments. For instance, urinary biomarkers can validate self‐reported data from questionnaires, addressing potential discrepancies, such as those observed in specific groups (e.g., the fluoridated milk group). This dual‐method approach could help identify inaccuracies in reporting and account for biological factors influencing fluoride absorption, ultimately improving public health evaluations of fluoride exposure risks.

The present study evaluated agreement and classification consistency between questionnaire‐ and urine‐based methods for assessing fluoride exposure but did not quantify the potential bias introduced by disagreement between methods, such as misclassification rates in epidemiological contexts. Addressing this limitation through formal modelling could provide important insights into the impact of method selection on exposure–outcome associations. Although the combination of both methods was proposed as a potential strategy to improve classification accuracy and reduce uncertainty, this approach was not examined analytically. Future research should investigate whether integrated methodologies offer measurable advantages in exposure assessment.

## 6 Strengths and Limitations

This study has several strengths. It is the first to directly compare two commonly used methods (24‐h urine sampling and a validated fluoride intake questionnaire) for estimating total daily fluoride intake in children across different fluoridation modalities. Paired data were collected from the same participants, enabling a direct within‐subject comparison. To enhance the quality of urine data, we applied urinary flow rate calculations to assess the completeness of the 24‐h collections and minimise underestimation due to missed voids. Additionally, dietary data were obtained through structured parent interviews conducted by trained personnel, which helped to ensure the accuracy and completeness of food and beverage intake records. These measures improved data reliability and strengthened the validity of the method comparison.

Nonetheless, several limitations should be acknowledged. Despite measures to evaluate urine completeness, it remains difficult to fully guarantee accurate 24‐h collection in young children due to practical challenges such as missed voids, enuresis, or limited cooperation. While structured interviews improved dietary reporting, the questionnaire method still relied on parental recall, which is subject to bias, particularly in estimating fluoride intake from toothpaste or unsupervised food consumption. Further, cross‐country differences in dietary habits and limited fluoride composition data for certain foods introduce uncertainty into the intake estimates. Although these limitations are largely inherent to the methods themselves rather than specific to this study's design, they underscore the complexity of accurately measuring fluoride exposure and interpreting discrepancies between assessment approaches.

## 7 Conclusion

The choice of fluoride intake assessment method should be guided by the specific objectives of the study and the characteristics of the target population. Given the observed discrepancies and limited agreement at the individual level between questionnaire‐based and urinary biomarker methods, relying on a single approach may lead to misclassification and inconsistent exposure estimates. Therefore, a combined strategy that integrates both questionnaire data and urinary biomarkers is recommended to improve the accuracy and reliability of fluoride intake assessments, enabling a more comprehensive understanding of exposure sources and potential health implications.

## Author Contributions

**F.V.Z., M.A.R.B., A.M., R.A.G**. and **S.M**. contributed to conception, design, and data interpretation; **F.V.Z., M.A.R.B., R.A.G**. and **S.M**. supervised the project at their respective sites; **R.S**. contributed to design, supervised data analysis and interpretation; **F.V.Z**. and **J.K**. conducted all data analysis and contributed to data interpretation, **K.G**. and **E.O.B**. supervised sample and data collection and contributed to data interpretation; **F.E**. and **F.M.L**. collected all data and samples at their respective sites; **F.V.Z**. drafted the manuscript. All authors critically revised the manuscript, gave their final approval and agreed to be accountable for all aspects of the work.

## Ethics Statement

All procedures were performed in compliance with relevant laws and institutional guidelines and have been approved by the appropriate institutional committees, including Teesside University, School of Health and Life Sciences Research Ethics Committee (#007/19, Oct/2019) in the UK, the Ethics Committee of Sao Paulo University for the study site in Bauru/Brazil (# CAAE 12565319.9.0000.5417, Jul 2019), the Ethics Committee of the University of Talca (#05‐2020E, Sept/2023) in Chile and the Administrative and Research Board of the Universidad El Bosque (UEB‐558, Sep/2020) in Colombia. The privacy rights of human subjects have been observed and written informed consent was obtained from the parent/legal guardian of the children recruited, prior to the study.

## Conflicts of Interest

The authors declare no conflicts of interest.

## Data Availability

The data that support the findings of this study are available from the corresponding author upon reasonable request.
